# Newly diagnosed ANCA-associated vasculitis after COVID-19 infection: a case report

**DOI:** 10.1186/s13256-023-04081-y

**Published:** 2023-08-26

**Authors:** Kazuhiro Kitamoto, Yasushi Tanaka, Tomohiko Kuboyama, Youhei Fujiki, Kodo Tomida, Takao Kamimori, Shigeo Hara

**Affiliations:** 1 Department of General Internal Medicine, Yodogawa Christian Hospital, 1-7-50 Kunijima, Higashiyodogawa, Osaka, Japan; 2 Department of Rheumatology, Yodogawa Christian Hospital, Osaka, Japan; 3 Department of Nephrology, Yodogawa Christian Hospital, Osaka, Japan; 4 Department of Diagnostic Pathology, Kobe City Medical Center General Hospital, Kobe, Japan

**Keywords:** COVID-19, ANCA-associated vasculitis, MPO-ANCA

## Abstract

**Background:**

Antineutrophil cytoplasmic antibody (ANCA)-associated vasculitis is a systemic autoimmune disease characterized by mononuclear cell infiltration and small and medium-sized blood vessel destruction leading to renal failure. Severe acute respiratory syndrome coronavirus 2 (SARS-CoV-2) has been shown to have the potential to induce the presentation or exacerbation of autoimmune disease. This report describes the clinical features of a case of newly diagnosed ANCA-associated vasculitis after COVID-19 Infection.

**Case presentation:**

During the COVID-19 pandemic, a 67- year-old female Japanese was undergoing treatment for interstitial pneumonia, diabetes mellitus, and hypertension at her local doctor. About 2 months ago, she was diagnosed with COVID-19 and went to a hotel for treatment, and her condition improved. But a month later, after her COVID-19 infection, she presented with a fever and cough and visited Yodogawa Christian Hospital in Osaka, Japan. The reverse transcription-polymerase chain reaction was negative. She underwent extensive radiological and laboratory investigations. Serologies revealed a high perinuclear-ANCA titer with a specific anti-myeloperoxidase antibody titer of 31.7 units/mL. We suspected ANCA-associated vasculitis and performed a renal biopsy. Renal biopsy showed evidence of crescentic glomerulonephritis, which was consistent with ANCA-associated vasculitis. The patient was referred to the Department of Rheumatology and Clinical Immunology for steroid pulse and cyclophosphamide treatment.

**Conclusions:**

Delayed screening may lead to progression of the autoimmune disease, so prompt diagnosis is necessary. In this case, we could make an immediate diagnosis and refer the patient to the Department of Rheumatology and Clinical Immunology.

## Introduction

Antineutrophil cytoplasmic antibody (ANCA)-associated vasculitis is a systemic autoimmune disease characterized by mononuclear cell infiltration and small and medium-sized blood vessel destruction leading to renal failure. Vasculitis is a descriptive term for a wide variety of conditions characterized by inflammation of the blood vessels that may occur as a primary process or secondary to an underlying disease. On the other hand, The novel coronavirus disease of 2019 (COVID-19) caused by severe acute respiratory syndrome coronavirus 2 (SARS-CoV-2) was first identified in Wuhan, Hubei, China, in December 2019 and resulted in a pandemic [1]. While systemic inflammation and pulmonary complication can result in significant morbidity and mortality, vasculitis may also occur [2]. This report describes the clinical features of newly diagnosed ANCA-associated vasculitis after COVID-19 Infection.

## Case presentation

A 67- year-old female Japanese was undergoing treatment for interstitial pneumonia, diabetes mellitus, and hypertension at her local doctor. About two months ago, she had a low-grade fever, tested positive for SARS-CoV-2 antigen, and was diagnosed with COVID-19. After that, she went to a hotel for treatment, and her condition improved. However, after COVID-19 infection, the patient had a low-grade fever for more than a month, and newly developed general malaise and dysesthesia in the left upper extremity. Despite treatment with azithromycin 500 mg daily, her condition did not respond to the medication. Therefore, she visited Yodogawa Christian Hospital in Osaka, Japan, requesting an alternative treatment. The reverse transcription-polymerase chain reaction was negative. She had no history of smoking. On physical examination, her vital signs were stable, with a blood pressure of 109/61 mmHg, a heart rate of 101 beats per minute and a temperature of 98.6℉. Her neck was supple. Her cardiovascular examination was regular, her lungs were clear to auscultation, and her abdominal examination was unremarkable, with no hepatosplenomegaly. A neurological examination showed dysesthesia in her distal left upper extremity and an inability to sense vibration. Skin findings were purpura on the right sole. She was admitted to the hospital for further examination and treatment.

Laboratory findings revealed an elevated white blood cell count of 215 × 10^2^/μL and elevated C-reactive protein 15.82 mg/dL, and elevated d-dimer 5.73 μg/mL (Table 1). Chest computed tomography (CT) showed no new pneumonia image, although there were still signs of interstitial pneumonia. Head CT, head magnetic resonance imaging, contrast-enhanced CT of the trunk, and echocardiography showed no abnormal findings. A skin biopsy was performed for purpura on the right sole but showed no abnormal results. A nerve conduction study was conducted for dysesthesia in the left upper extremity, but no abnormal findings were found.

**Table 1:** Laboratory data on admission

Parameter	Recorded value	Standard value
Blood		
White blood cell count	215 × 10^2^/μL	32–85 × 10^2^/μL
Neutrophil	84.0%	
Lymphocyte	7.0%	
Eosinophil	1.0%	
Hemoglobin	10.0 g/dL	11.3–15.2 g/dL
Hematocrit	31.1%	36–45%
Platelet	44.6 × 10^4^/μL	13–34.9 × 10^4^/μL
C-reactive protein	15.82 mg/dL	≦ 0.29 mg/dL
Total protein	7.3 g/dL	6.5–8 g/dL
Albumin	2.1 g/dL	4-5.2 g/dL
Aspartate aminotransferase	24 U/L	0–30 U/L
Alanine aminotransferase	23 U/L	0–30 U/L
Lactate dehydrogenase	147 U/L	106–220 U/L
Creatine phosphokinase	19 U/L	62–287 U/L
Blood urea nitrogen	16.9 mg/dL	7–24 mg/dL
Creatinine	0.87 mg/dL	0–1 mg/dL
Sodium	132 mEq/L	136–147 mEq/L
Potassium	4.3 mEq/L	3.6–5.0 mEq/L
Glucose	141 mg/dL	70–99 mg/dL
Hemoglobin A1c	6.9%	4.6–5.5%
Rheumatoid factor	200 IU/mL	0–15 IU/mL
Cyclic citrullinated peptide antibody	0.6 U/mL	0–4.5 U/mL
Anti-Jo-1 antibody	1.0 U/mL	0–10 U/mL
Anti-myeroperoxidase antibody	31.7 U/mL	0–3.5 U/mL
Anti-proteinase 3 antibody	1.0 U/mL	0–3.5 U/mL
Urine	Qualitative protein 1+	Qualitative occult blood 2+

**Fig. 1:**
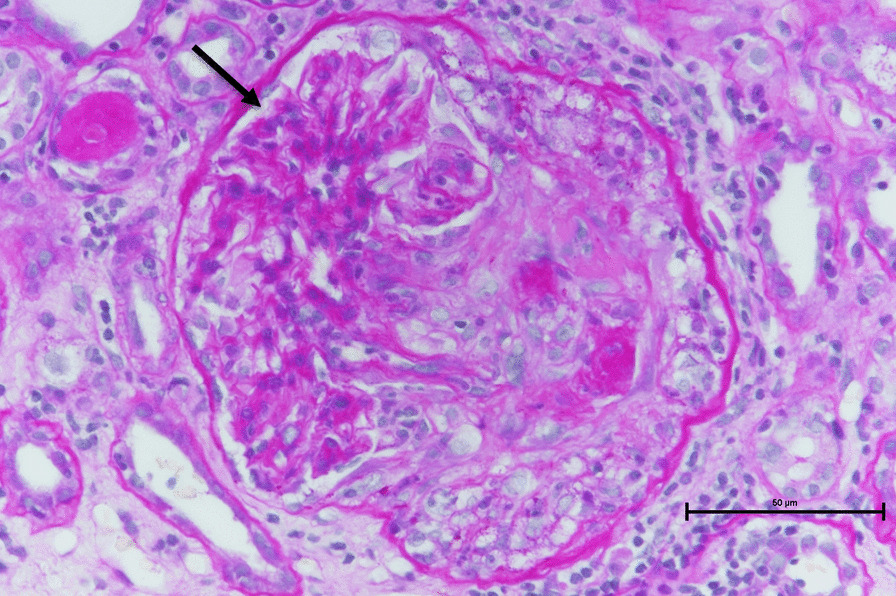
Renal biopsy showed evidence of crescentic glomerulonephritis (black arrow, periodic acid-Schiff stain)

After admission to the hospital, her temperature ranged between 98.6 ℉ and 100.4 ℉. Initially, we suspected infection as the cause of the inflammation, but since there were no significant findings on various imaging tests, we suspected autoimmune disease and measured different antibodies by blood tests; since high perinuclear-ANCA titer with a specific anti-myeloperoxidase antibody (MPO) titer of 31.7 units/mL, we suspected ANCA-related vasculitis and performed a renal biopsy. Renal biopsy showed evidence of crescentic glomerulonephritis (Fig. 1), which was consistent with ANCA-associated vasculitis. The patient was referred to the Department of Rheumatology and Clinical Immunology for steroid pulse and cyclophosphamide treatment. Steroid and cyclophosphamide pulses were initiated as induction remission therapy, and azathioprine 25–50 mg/day was added as maintenance therapy. During treatment, type 2 diabetes worsened with steroid administration and required insulin induction. These treatments improved inflammatory findings, MPO-ANCA decreased to 1.1 units/mL, and CT showed improvement in interstitial pneumonitis.

## Discussion and conclusion

There are three types of ANCA-associated vasculitis, which is an autoimmune disease: microscopic polyangiitis (MPA), granulomatosis with polyangiitis (GPA), and eosinophilic granulomatosis with polyangiitis (EGPA). With early diagnosis and treatment, good improvement can be obtained. Among ANCA-associated vasculitis, alveolar haemorrhage and interstitial pneumonia are seen in MPA, bronchial asthma and necrotizing granulomatous bronchopulmonary lesions are seen in EGPA, and pulmonary lesions such as granulomatous bronchopulmonary lesions and alveolar haemorrhage are seen in GPA. In Japan, the frequency of MPA is higher than in Europe and the United States, and interstitial pneumonia and alveolar haemorrhage are frequently observed. In a previous study, interstitial pneumonia was associated with 29–49% of MPO-ANCA-positive vasculitis in Japan [3, 4]. This is higher than the 11% reported in Europe and the United States and is considered a characteristic of vasculitis in Japan [5]. In many cases, interstitial pneumonia precedes the onset of vasculitis by several years, and occult urine blood is often observed several years after interstitial pneumonia. In this case, the screening done several years ago was negative for antibodies to any autoimmune disease, including ANCA. Patients with a fever of interstitial pneumonia should be tested for COVID-19 but also checked for interstitial and new pneumonia exacerbations. In addition, it is crucial to screen for autoimmune diseases such as rheumatoid arthritis, polymyositis/dermatomyositis, and ANCA-associated vasculitis. Delayed screening may lead to the progression of the autoimmune disease, so prompt diagnosis is necessary. In this case, we could make an immediate diagnosis and refer the patient to a specialized department.

It is interesting in the association of COVID-19 and ANCA-associated vasculitis that SARS-CoV-2 infection can trigger vasculitis; SARS-CoV-2 infection may trigger autoimmunity and autoimmune diseases [6, 7]. Previous studies have reported systemic viral and bacterial infections can induce autoimmunity and cause GPA. [8, 9]

Mechanisms that induce autoimmunity after SARS-CoV-2 infection include bystander killing, molecular mimicry, viral persistence, epitope spread, and neutrophil extracellular trap formation [10, 11]. In addition, the pathophysiology of COVID-19 still needs to be fully understood and continues to be studied; the symptoms it may cause in patients with established or new-onset ANCA-associated vasculitis still need to be discovered.

## Data Availability

Not applicable.

## Abbreviations

ANCA: Antineutrophil cytoplasmic antibody
COVID-19: Coronavirus disease of 2019
SARS-CoV-2: Severe acute respiratory syndrome coronavirus 2
CT: Computed tomography
MPO: Anti-myeloperoxidase antibody
MPA: Microscopic polyangiitis
GPA: Granulomatosis with polyangiitis
EGPA: Eosinophilic granulomatosis with polyangiitis
